# Listening to a conversation with aggressive content expands the interpersonal space

**DOI:** 10.1371/journal.pone.0192753

**Published:** 2018-03-28

**Authors:** Eleonora Vagnoni, Jessica Lewis, Ana Tajadura-Jiménez, Flavia Cardini

**Affiliations:** 1 Department of Robotics, Brain and Cognitive Sciences, Istituto Italiano di Tecnologia, Center for Human Technologies, Genoa, Italy; 2 Department of Psychology, Anglia Ruskin University, Cambridge, United Kingdom; 3 Interactive Systems DEI-Lab, Universidad Carlos III de Madrid, Madrid, Spain; 4 UCL Interaction Centre (UCLIC), University College London, London, United Kingdom; Centre de neuroscience cognitive, FRANCE

## Abstract

The distance individuals maintain between themselves and others can be defined as ‘interpersonal space’. This distance can be modulated both by situational factors and individual characteristics. Here we investigated the influence that the interpretation of other people interaction, in which one is not directly involved, may have on a person’s interpersonal space. In the current study we measured, for the first time, whether the size of interpersonal space changes after listening to other people conversations with neutral or aggressive content. The results showed that the interpersonal space expands after listening to a conversation with aggressive content relative to a conversation with a neutral content. This finding suggests that participants tend to distance themselves from an aggressive confrontation even if they are not involved in it. These results are in line with the view of the interpersonal space as a safety zone surrounding one’s body.

## Introduction

The space close to our body is particularly important given that it is where we physically interact with stimuli in the external world. Several disciplines have investigated this space using different paradigms and terminology. In social psychology, ‘personal space’ is often used to define the emotionally-tinged zone around the human body that people experience as ‘their space’ [1] and which others cannot intrude without causing discomfort [2]. This definition suggests that personal space only exists during interaction with other people [3]. Hence, the terms ‘interpersonal space’ (IPS) and ‘interpersonal distance’ (IPD), which define the space/distance individuals maintain between themselves and others, are often used as synonyms of ‘personal space’ [4, 5]. In the cognitive neuroscience tradition, instead, this space has been referred to as ‘peripersonal space’ (PPS) [6] and has been defined as the space immediately surrounding our body. The discrete coding of peripersonal space in the brain was first revealed by single-cell recordings in monkeys, within a network of interconnected sensori-motor areas [7, 8, 9, 10, 11]. Later, neuroimaging and neurophysiological studies showed a similar fronto-parietal network in humans [12, 13, 14, 15, 16, 17]. Cognitive neuroscience studies have focused mainly on two interpretations of PPS, i.e. a space for action and a defensive space. According to the first interpretation, the PPS is where goal-directed actions occur, and where objects can be grasped and manipulated, while objects located beyond this space cannot [18, 19]. According to the second interpretation, the PPS is the space that we maintain between our body and dangerous objects, like a protective bubble that keeps a margin of safety around the body surface and coordinates defensive behaviours against potentially dangerous stimuli [20, 21, 22]. Recently, a dual model of PPS has been proposed, which is based on a functional distinction between defensive and goal-directed action space [23]. According to this model the two functions of PPS require distinct sensory and motor processes. For example, the goal-directed PPS, generally, requires finer and more controlled motor actions than the defensive PPS, while defensive actions are frequently, but not always, automatic.

Moreover, different factors seem to influence the two types of PPS and sometimes the same factor modulates them in opposite ways. For example, whereas anxiety expands the defensive PPS [24, 25] it shrinks the goal directed PPS [26]. Indeed, in the first case the safety bubble around us becomes bigger and objects that are normally considered innocuous, as far from our body, are instead treated as within our PPS boundaries. In the mentioned studies the ‘size’ of the PPS was measured with the bisection line paradigm [24] and the hand-blink reflex (HBR) [25]. The line bisection paradigm measures the bias in a visual bisection task. When bisecting horizontal lines close to the body observers show a slight leftward bias that, however, shifts rightward when the line is presented in far space [27] (see [28] for review). Lourenco and colleagues [24] found that subjects with high level of claustrophobia showed a more gradual rightward shift over distance. The authors interpreted the results as evidence of a larger representation of their PPS space due to their claustrophobia-related anxiety [24]. In the hand-blink reflex (HBR), instead, participants receive a stimulation on the median nerve that produce an eye blink reflex when the hand is located close to the face [29, 30]. With this paradigm the authors showed that in more anxious individuals, the “safety margin” is located further away from the body than in less anxious individuals. In this case the boundaries of PPS are measured with the strength of the HBR in relation to the position of the stimulated hand from the face.

Conversely, anxiety seems to reduce the size of our goal directed PPS, as it has been shown that anxious people perceive themselves as less able to perform a movement [26]. In this case participants were asked to judge if they were able to reach an object on a table. When anxiety was experimentally induced participants underestimated their ability to reach for the (inoffensive) objects on the table.

Both the social psychology and cognitive neuroscience literature have shown that people’s mental representation of the space around their body is not fixed. Experimental evidence from cognitive neuroscience studies showed an expansion of the PPS representation after tool use: when, through a tool, people act upon far space their representation of near and far space changes, with the far space being remapped as near space [31, 32, 33]. This effect was first described in monkeys, indeed, Iriki and colleagues [34] analyzed the responses of neurons in the post-central gyrus of the monkey after training the monkey to reach food with a rake. Interestingly, after the training neurons in the post-central gyrus responded to visual stimulation in the monkey’s extrapersonal space. According to the authors, the visual receptive fields of neurons representing the PPS expanded following tool use [34]. Similarly, in humans, Canzoneri and colleagues [35] showed that a brief training with a tool induces plastic changes both to the representation of the body part using the tool and to the PPS. Interestingly, not only the hand-centered PPS expands after training with tools. Indeed, Galli and colleagues [36] have shown the effects of a special tool, the wheelchair, in extending the action possibilities of the whole body. Even if the hand-centered PPS is the most investigated, there is evidence of at least three body-part specific PPS representations, face, hand and trunk-centered, that differ in extension and directional tuning [37]. These experiments used several versions of a well-validated bimodal paradigm [35, 36, 37, 38, 39]. In this paradigm sounds approaching the participant’s body are presented while tactile stimuli are delivered to the participant’s hand at several time delays. Specifically, the tactile stimulus is delivered when the sound is perceived at several distances from the body. The participants are asked to respond as quickly as possible to the tactile stimulus ignoring the approaching sound. It has been shown that the reaction times to the tactile stimuli are modulated by the simultaneous presentation of the to-be-ignored sound. Indeed, the reaction times become progressively faster as the sound is perceived closer to the body. This paradigm allows identifying the PPS boundaries, and quantifying their variation due to various factors (e.g., after tool use, as in [35]). Moreover, the same paradigm has been used to show how multisensory inputs, even outside of awareness, are integrated within the PPS [40].

The expansion of PPS after tool use relates to the interpretation of PPS as the space where goal-directed actions occur. The PPS representation seems to expand also in the presence of unpleasant or threatening stimuli, which relates to the interpretation of PPS as a protective space: when a threatening stimulus is approaching our body, we expand our safety zone [41, 42, 43, 44]. It has been suggested that coding a dangerous stimulus as inside our safety zone earlier than a non-dangerous stimulus has an adaptive advantage given that it allows having more time to engage in a defensive response [41].

Recently the influence of social interaction on PPS has been investigated also in cognitive neuroscience studies. Indeed, it has been shown that PPS boundaries shrink when subjects sit in front of another person, as compared to a mannequin, placed in far space [45]. Interestingly, after playing an economic game with another person, PPS boundaries between self and other merge when performing a task with another person, but only if the other person behaves cooperatively [45]. Moreover, a recent study demonstrated that shared sensory experiences between two people induced by interpersonal multisensory stimulation, do not only increase the remapping of the other’s sensory experiences onto the participant’s own body [46] but also alter the way in which PPS is represented [47]. In particular, by using the bimodal paradigm previously described [35, 36, 37], Maister and colleagues showed a significant increase in audio-tactile integration in the space close to the confederate’s body after the shared experience [47]. These results suggest that sharing multisensory experiences can induce a remapping of the other’s PPS onto our own PPS [47]. In relation to shared PPS, Brozzoli and colleagues have identified neuronal populations in the human ventral premotor cortex that encode the space near both one’s own hand and another person’s hand. This suggests that we use a common spatial reference frame to code sensory events, actions and cognitive processing happening within the shared PPS [48].

Social psychology studies showed how people tend to react to spatial violations by increasing distance from intruders when feeling in hostile and uncomfortable situations and, vice-versa, by reducing distance when feeling in friendly and comfortable situations [49, 21]. A typical task to assess the size of IPS is based on comfort-distance judgments provided through the ‘stop-distance’ paradigm: in this task participants are required to stop the person walking towards them when they start to feel uncomfortable with the other’s proximity (passive ‘stop-distance’ task; [2, 50, 51, 52, 53, 54]) or have to walk towards a person and stop themselves when they start to feel uncomfortable with the other’s proximity (active ‘stop-distance’ task; [33, 54, 55, 56]). With this paradigm, it has been shown that the size of IPS is modulated by the situational, emotional and individual characteristics [2, 51, 57]. For example, listening to positive versus negative emotion-inducing music reduces the representation of IPS, allowing others to come closer to us [54]. Moral information about the confederates modulates the IPS as well, so that participants choose to increase the distance between themselves and an immorally described confederate while they reduce the distance with a morally described confederate [55].

From an ‘action-centered’ perspective, IPS can be seen as the physical space where social interactions occur [56]. Lloyd and Morrison [58] suggested that the nature of the social interactions, and the characteristics of the person we are interacting with, may affect IPS. In their study, using fMRI they measured brain activity while participants viewed photographs where one person either posed a potential threat to another (threat condition) or it did not pose a threat (non-threat condition). Crucially, the two people were depicted close or far from each other. The temporal–occipital junction, extrastriate, and fusiform cortices and right superior parietal lobe (BA7)—which are visuospatial areas—responded when the threatening person was close to the other person (in this case the authors used the term ‘personal space’), but not when the two people were distant from each other. From these results the authors concluded that higher-level visual cortices seemed to play a role in distinguishing social categories based on a person’s features, e.g. how dangerous a person looks, and that it is not only the presence of a personified threat, but the spatial distance between the people interacting, that together influences an observer’s interpretation of the interaction. Moreover, results showed that posterior parietal areas—which code the space surrounding one’s own body (see [59])—responded when the individuals were closer, regardless of whether the person was depicted as threatening or not. Observing interactions in which one is not directly involved seemed to influence one’s own IPS. The authors referred here to the term ‘eavesdropping’ that ethologists use to describe the process of gaining relevant information on an individual—such as status, aggression potential or sexual desirability- by observing him/her interacting with others [60]. This information allows preparing for action before a direct interaction takes place, which may be especially important in situations which pose a potential threat to one’s body.

The concept of ‘eavesdropping’ is particularly relevant in the present study. Indeed, we aimed to directly investigate the influence that the interpretation of other people interaction, in which one is not directly involved, may have on a person’s IPS. In particular, we hypothesized that the emotional content of a heard conversation will modulate the participant’s IPS representation, even if the participant is not involved in that conversation. In this experiment, participants listened to two different conversations between two persons, one conversation had an aggressive content and the other had a neutral content. After listening to each conversation, the comfort IPS of participants was measured by using the ‘stop-distance’ paradigm, which was described above and that it has been widely used to investigate the IPS representation [2, 50, 51, 52, 53, 54]. Usually in this paradigm the participants actively approach, or are approached by, another person that they are looking at. Here we decided to measure the IPS when no visual information about the other person was available to the participants. Indeed, no one was in front of the participant but participants listened to the recording of the footstep sounds of a person walking towards them. They were asked to stop the recording as soon as they started feeling uncomfortable, or as the footsteps were perceived too close to them. Interestingly, this modified version of the more classic paradigm resulted to be effective in measuring the IPS. Using the sound of approaching footsteps has the advantage of eliminating any interactions and confounds with the idiosyncrasy of the person approaching (actively or passively) the participants. Moreover, the content of the conversation modulated the IPS with participants stopping earlier the footsteps recording after listening to an aggressive conversation relative to a neutral one. Therefore, listeners seem to distance themselves more from someone approaching after a conflictual discussion.

## Method

### Participants

The present research involved human participants and has been approved by the local ethical committee–i.e. the Faculty Research Ethics Panel, at Anglia Ruskin University—and has been conducted according to the principles expressed in the Declaration of Helsinki. Written informed consent was obtained from the participants.

Thirty-three participants (21 female) between 19 and 30 years of age, mean age 21.7 took part in the experiment. They were members of the Anglia Ruskin community and participated in the experiment in exchange for course credit. Participants reported normal or corrected-to-normal vision.

### Stimuli, design, and procedure

A pair of binaural microphones (Core Sound, frequency response 20 Hz-20 kHz) and an audio recorder (ZOOM ZH4N Handy Portable Digital Recorder) were used to record the sound stimuli used in the experiment. Specifically, the sound of “approaching footsteps” (i.e., the sounds produced by a person walking towards the listener) were recorded in an empty and quiet large room with a length of 17.4 m and wooden floor. The recorder was placed at one end of the room and the “walker”, a female wearing hard-sole shoes, positioned herself at the opposite end of the room. She was instructed to walk towards the recorder, at a natural gait speed and keeping a constant pace. This produced an “approaching footsteps” recording that lasted 42.59 seconds. In order to ascertain that the footsteps sound was perceived as approaching, we asked 20 participants–who did not take part in the main experiment–to listen at the recording and rate the direction of the sound on a Likert scale from -5 (“receding”), to +5 (“approaching”) with 0 as “walking in place”. Participants’ ratings were all 4 or above (median = 5, range = 4–5) and every participants reported that the sound was clearly perceived as approaching their body.

The two conversations were performed by two actors, one male and one female, both drama students at Anglia Ruskin University (both actors gave informed consent before being recorded). The conversations were not scripted, the actors improvised from selected topics: first date, catch up between old friends, infidelity within a relationship and drunk fight. Two researchers independently chose the best recording for each condition, and they coincide on the ‘first date’ for the neutral and ‘drunk fight’ for the aggressive condition. The conversations were recorded in an empty and quiet corridor of the same length as the room where the footsteps were recorded, the recorder was placed at one end of the corridor and the actors positioned themselves at the opposite end. The conversations’ audio clips lasted 165 seconds.

**Fig 1:**
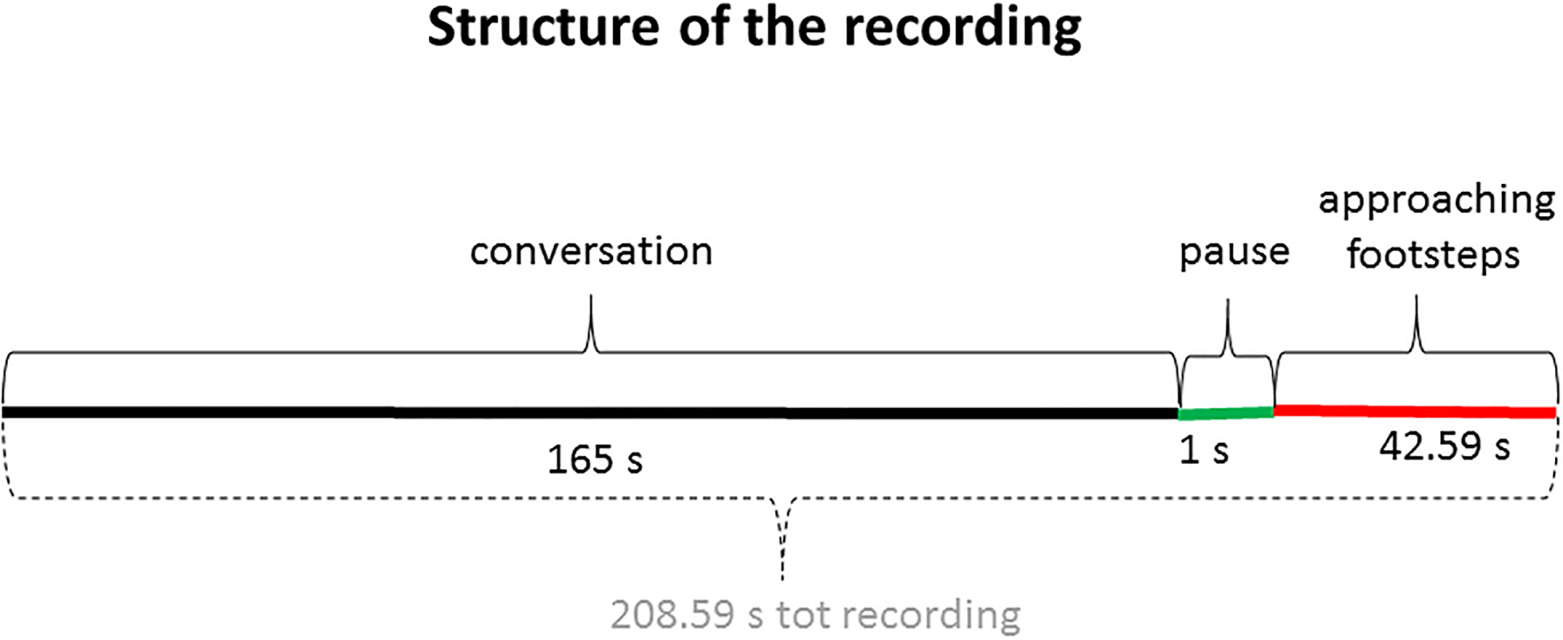
Structure of the recording. The recording was created concatenating two recordings (conversation and footsteps), leaving 1 second silence gap in between. The recording of the conversation lasted 165.00 seconds (for both aggressive and control condition). This was followed by a silence pause lasting 1 second, after which the recording of the footsteps started. The footsteps recording lasted 42.59 seconds—this was the time window that the participants had to respond.

We merged the audio clips of the conversations with the “approaching footsteps” clip with Audacity 2.1.2 Software leaving one second gap between the end of the conversation and the beginning of the footsteps. These produced two experimental stimuli, one used for the neutral condition and one used for the aggressive condition. For each condition, the resulting audio clip lasted a total of 208.59 seconds. Therefore, there were 165.00 seconds of conversation, 1 second of pause and 42.59 seconds of approaching footsteps (Fig 1). During the last 42.59 seconds, participants could either produce a response by stopping the recording, if they felt uncomfortable, or let the recording play until its end, if they did not feel uncomfortable.

The stimuli were presented through a Tablet (Acer Aspire Switch 10) using Audacity 2.1.2 Software. Participants were asked to wear a blindfold and noise cancelling headphones. All participants listened to both the aggressive and neutral conversations, in a counterbalanced order. The sound level was approximately 65 dBA. After each conversation ended, the participants heard footsteps approaching them. They were asked to press the keyboard key “P” when they felt like the footsteps were too close and started to make them feel uncomfortable. In the case the footsteps did not make participants feel uncomfortable they could just let the recording of the footsteps play until its end.

## Results and discussion

**Fig 2:**
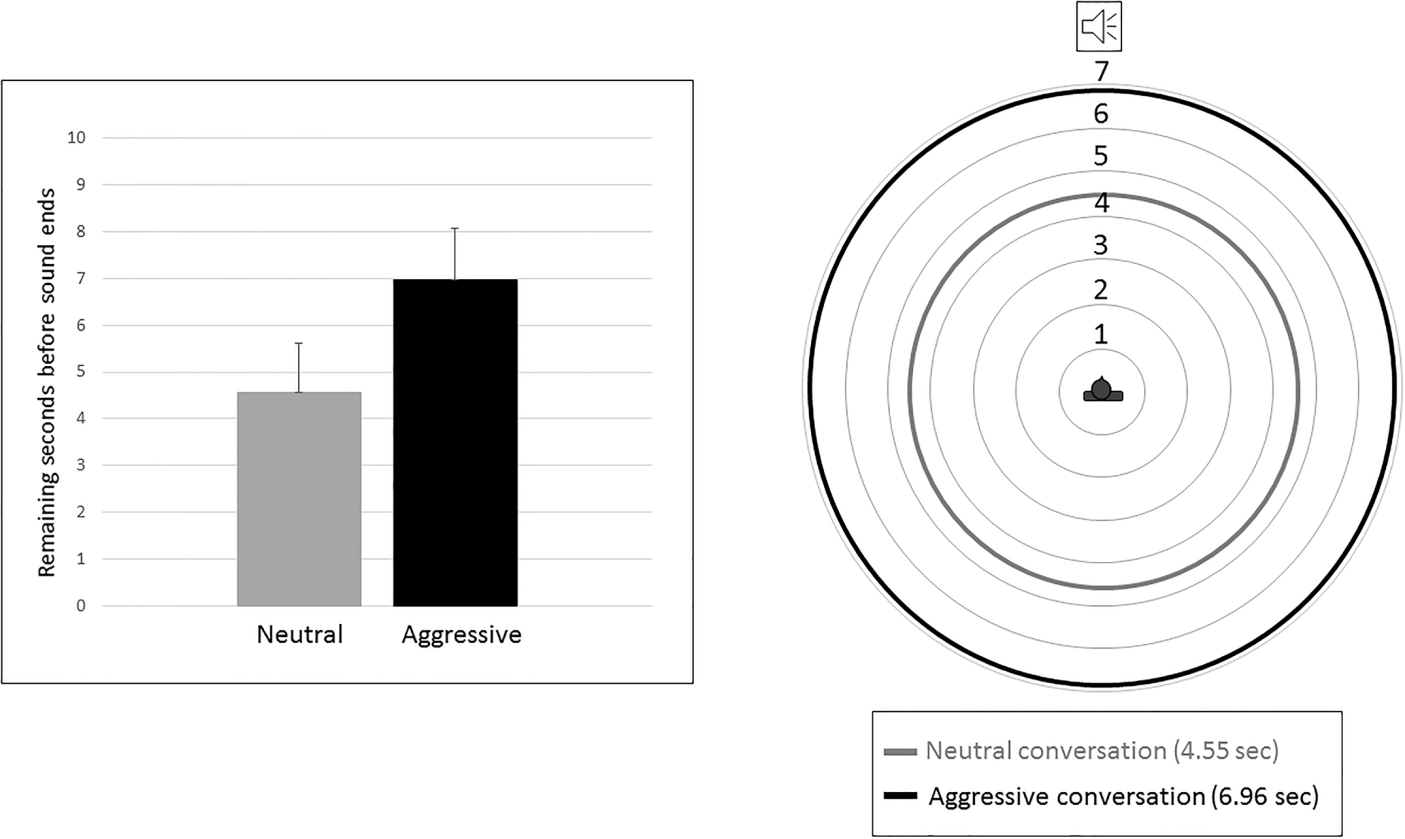
The left panel shows the average time (and standard error) at which participants stopped the footsteps recording for the neutral condition (M 4.55 s, SE 1.06 s) and aggressive condition (6.96 s, SE 1.13 s). The right panel shows how the participants’ responses would be translated in the space domain.

We subtracted the response time of the participants (when they pressed the keyboard key) from the total duration of the recording (208.59 s), with higher values indicating that the participants stopped the recording sooner (S1 File). In the aggressive condition (M = 6.96 s, SE = 1.13 s) participants stopped the recording after 201.63 seconds while in the neutral condition (M = 4.55 s, SE = 1.06 s) after 204.04 seconds (Fig 2). To put it in simpler words, since the time window available for the participants to give their response was the 42.59 seconds of approaching footsteps, this means that in the aggressive condition the footsteps were stopped after 35.04 seconds and in the neutral condition after 37.45 seconds, demonstrating that participants started feeling uncomfortable sooner in the first condition–i.e. after listening to an aggressive conversation—than in the second–i.e. after the neutral conversation.

A Kolmogorov-Smirnov test was used to test the data for normality. RTs for both conditions were not normally distributed (p < .05). Therefore, we used non-parametrical statistical tests to analyze the data.

As all participants listened to both the aggressive and neutral conversations, in a counterbalanced order, we first ran a Kruskal-Wallis test to compare RTs in each condition (neutral and aggressive conversation) across the two groups of participants (i.e. those who listened to the neutral conversation first vs those who listened to the aggressive conversation first), in order to test whether the order of the conversation presentation had any impact on the RTs. Results showed that RTs in the neutral conversation did not significantly differ between the two groups: X2(1, N = 33) = 1.13, p = .28. Similarly, RTs in the aggressive conversation condition did not significantly differ between the two groups: X2(1, N = 33) = .29, p = .58. Therefore, the type of conversation that participants listened first did not affect the time at which participants interrupted the “approaching footsteps’” clip.

Second, we ran a Friedman test to compare the RTs after the two conversations (neutral vs aggressive). Results showed that participants stopped the “approaching footsteps” clip earlier after listening to an aggressive conversation (M = 6.96 s, SE = 1.13 s) compared to after listening to a neutral conversation (M = 4.55 s, SE = 1.06 s): X2(1, N = 33) = 8.53, p = .003. The present results showed that the mere sound of approaching footsteps, instead of visual information of another person, can be used to measure the IPS using the ‘stop-distance’ paradigm. Indeed, with this study we have shown for the first time that it is possible to measure the IPS representation even without an actual person standing in front of the participants. We presented a recording of approaching footsteps and asked the participants to stop the recording when the distance from the walker was making them feeling uncomfortable. This modified version of the more classic ‘stop-distance’ task is not only effective but it also eliminates any possible influence of the idiosyncrasy of the person standing in front of the participants.

Interestingly, after listening to a conversation with an aggressive content the participants stop the sound of approaching footsteps further away from their body relative to after listening to a conversation with neutral content. Therefore, after listening to an aggressive conversation the IPS representation increases with participants setting a wider distance between them and the approaching footsteps. This increased distance could be interpreted as an attempt to avoid being involved in an aggressive confrontation or to avoid an interaction with a person soon after a threatening social interaction. The results showed that 11 participants in the control condition and 6 participants in the experimental condition did not stop the recording. On one hand this result points towards the fact that participants who stopped the recording did so because they felt uncomfortable and, on the other, that more people in the experimental condition did feel uncomfortable (more people stopped the recording) therefore showing the effectiveness of our manipulation.

Our result is consistent with previous findings from the social psychology literature [61, 62]. For example, Lieberz and colleagues [61] showed that women detect implicit cues of aggressiveness in male faces and adjust their interpersonal distance behaviour accordingly. Moreover, a recent study found an increased interpersonal space when an angry confederate (virtual character) approached participants [62]. Participants tend to maintain a certain distance from threatening stimuli approaching the body as self-protection response [49, 55, 63, 64]. Interestingly, we showed for the first time that the quality of social interactions influences our comfort boundaries even when we are not directly involved in the aggressive confrontation.

In this study we have shown how being the bystander of an aggressive confrontation influences the distance that we take from other people. Perceived threat from others represents a crucial factor in mediating the equilibrium between interpersonal space and social interaction [50, 56, 65, 66]. Moreover, information gained from observing, or in this case listening to, human interactions may contribute to an evaluation of both the situation and the person involved and thereby facilitates social learning particularly in fearful or threatening situations [56, 67].

In the cognitive neuroscience literature, many studies focused on the defensive aspect of PPS [20, 42, 43, 44]. However, rarely the threatening objects used in these studies were social. On the other hand, the social psychology literature focused on the distance between two people but rarely the interacting person was represented as threatening. The study of Lloyd and Morrison [58] is relevant to the present work given that showed a network of areas involved in interpersonal spatial behaviour that is modulated not only by the distance between the interactants but also by the nature of the interaction. Moreover, as in our case, the participants were not directly involved in the social interaction but they were observing the interaction between two people. We believe that the IPS can be interpreted, as it has been done for the PPS, as a margin of safety around the body. However, it is difficult to exactly say if IPS and PPS are just two terms used to indicate the same portion of space [55, 56] or if they are two functionally different and independent spatial representations [33, 68]. Patané and colleagues [33] compared the effect of tool use on reaching distance and comfort distance and found an effect of tool use on the reaching distance but not comfort distance. Moreover, in a following study [68] the authors showed dissociation between PPS, operationalized as reachable space, and IPS, operationalized as comfort space. Specifically, the authors used a ‘social’ tool-use setting in which tools were not only bodily extensions, but instruments for social cooperation. Indeed, the participants had to cooperate, using the tools, to complete the task. The results showed an effect of cooperative tool use on PPS but not on IPS. It would be interesting to investigate if the defensive PPS and IPS have the same constrains. Although one might argue that the notion of IPS as ‘comfort zone’ is closer to the ‘margin of safety’ interpretation of PPS. It is indeed impossible to feel comfortable when not safe.

Both PPS and IPS have been investigated in relation to anxiety and social disorders. As already mentioned, the size of PPS is modulated by the anxiety level of the individuals, with more anxious individuals showing a larger PPS [29, 30]. Moreover, it has been shown that individuals with higher level of claustrophobia show a larger and less flexible PPS [24, 69]. Regarding socio-communicative disorders several works have focused instead on the IPS. Specifically, the IPS of individuals with persistent difficulties has been investigated in the domain of social behavior, such as children with autism spectrum disorders (ASD). Using the stop-distance paradigm it has been shown that ASD children feel comfortable at a greater distance relative to children with typical development [70] (but see also [71] and [72] for different results on the size of PPS). Moreover, ASD children seems to have a less flexible IPS [73], in accordance with [71] and [72]. This last finding is in agreement with the hypothesis that ASD children show a steeper and less flexible gradient between self and other [74]. Indeed, it has been proposed that schizophrenia and ASD can be considered two extremes of an element of self-representation, self-location [74], and, specifically, the distance between self and other.

Our modified version of the stop-distance task and the manipulation used in this study could be helpful to investigate the IPS in individuals with socio-communicative disorders or high level of social anxiety. It would be interesting, for example, to investigate if individuals with high social anxiety show flexibility of their IPS or if the mere presentation of a conversation, even with a neutral content, is enough to make them feel uncomfortable and avoid the cue of an approaching person (i.e. approaching footsteps).

Future work may also compare the different effects on IPS of using visual and auditory stimuli in ‘stop-distance’ paradigms. Visual and auditory systems differ in their processing of environmental signals. When considering the IPS as a ‘comfort zone’ or a ‘margin of safety’, one could hypothesize that the auditory stimuli may have a greater influence on IPS, as it is indeed being categorized as a ‘warning system’ (e.g., [75]). This categorization derives from a number of advantages that the auditory system displays, as compared to the visual and other sensory systems. These include being characterized as a ‘change detector’, with high temporal resolution and high sensitivity for structured motion that allow to quickly extract cues indicating a rapid change and quickly orient behaviour towards it, in a faster way than the visual system [75, 76]. A second advantage is that audition provides a continuous stream of information on distant and close stimuli–while we regularly block vision by closing our eyes, our ears cannot be ‘turned off’ in the same way [77]. Finally, audition informs about events taking place all around us, even those events outside the visual field, and process several streams of information in parallel, which provides and overall impression of the events around us, as well as an impression of the geometry and size of the space we are in, through the acoustic reflections with the surrounding objects and walls [78, 79]. Given these differences in processing of information, it may be hypothesized than the size of the IPS, as measured by ‘stop-distance’ and other paradigms, may be modulated differently by visual and auditory stimuli. Future work could test this hypothesis, while also considering that in complex real-life contexts we often encounter a combination of information from different sensory modalities, which relate to different events. Our brain needs to monitor, integrate and respond to all these different cues in an optimum way that allows to keep us safe, and if possible, comfortable.

## Supporting information

S1 File Data. Data coded both in time (s) and distance (cm).
(XLSX)
